# Misalignment of global COVID-19 breastfeeding and newborn care guidelines with World Health Organization recommendations

**DOI:** 10.1136/bmjnph-2020-000184

**Published:** 2020-12-22

**Authors:** Duong Vu Hoang, Jennifer Cashin, Karleen Gribble, Kathleen Marinelli, Roger Mathisen

**Affiliations:** 1 Alive & Thrvie Southeast Asia, FHI 360, Hanoi, Vietnam; 2 Alive & Thrive Southeast Asia, FHI 360, Yangon, Myanmar; 3 Western Sydney University School of Nursing and Midwifery, Penrith South DC, New South Wales, Australia; 4 Pediatrics, University of Connecticut School of Medicine, Hartford, Connecticut, USA; 5 Alive & Thrive Southeast Asia, FHI 360, Hanoi, Vietnam

**Keywords:** malnutrition, mental health

## Abstract

**Introduction:**

Recommendations for the clinical management of new mothers with suspected or confirmed COVID-19 and their infants are required. Guidance must weigh the risk posed by transmission of SARS-CoV-2 against the protection that maternal proximity and breastfeeding provide infants. Our aim was to review international COVID-19 guidance for maternal and newborn care, assessing alignment with WHO recommendations and the extent to which policy supported or undermined breastfeeding.

**Methods:**

Guidance documents from 33 countries on the care of infants whose mothers were suspected or confirmed as having COVID-19 were assessed for alignment with WHO recommendations regarding: (1) skin-to-skin contact; (2) early initiation of breastfeeding; (3); rooming-in; (4) direct breastfeeding; (5) provision of expressed breastmilk; (6) provision of donor human milk; (7) wet nursing; (8) provision of breastmilk substitutes; (9) psychological support for separated mothers; and (10) psychological support for separated infants.

**Results:**

Considerable inconsistency in recommendations were found. Recommendations against practices supportive of breastfeeding were common, even in countries with high infant mortality rates. None of the guidance documents reviewed recommended all aspects of WHO guidance. The presence of influential guidance conflicting with WHO recommendations and an undervaluing of the importance of maternal proximity and breastfeeding to infant health appeared to contribute to this poor alignment.

**Conclusion:**

Those developing guidance in the COVID-19 pandemic and other infectious disease outbreaks need to appropriately consider the importance of skin-to-skin contact, early initiation of breastfeeding, rooming-in and breastfeeding to maternal and infant physical and psychological health. In weighing the value of recommendations of others in future guidance development, countries should consider past reliability and value placed on breastfeeding. Recommendations against maternal proximity and breastfeeding should not be made without compelling evidence that they are necessary, and less harmful than maintaining dyad integrity.

**Key questions**

**What is already known?**

- Interruption of exclusive and continued breastfeeding is responsible for nearly 700,000 maternal and child deaths annually.
- Concern about mother-to-infant transmission of SARS-CoV-2 in the COVID-19 pandemic has caused separation of mothers and newborns throughout the world, reducing breastfeeding.
- The WHO issued guidance for mothers suspected or confirmed as having COVID-19 and their newborns that supported maintaining mother and infants proximate to one another and early and exclusive breastfeeding.

**What are the new findings?**

- None of the guidance from the 33 countries included in our study recommended all aspects of WHO guidance.
- Most countries surveyed did not recommend keeping mothers and infants in close proximity or direct breastfeeding.
- It was uncommon to recommend psychological support for mothers and rare to recommend psychological support for infants, where mother and infant were isolated from one another because of COVID-19.

**What do the new findings imply?**

- Mothers and their newborns have been separated and breastfeeding impeded or prevented around the world because of concern regarding mother to infant transmission of SARS-CoV-2.
- Decisions related to maternal and newborn proximity and breastfeeding have been based on other prominent organisations whose early guidance were based on fear of the unknown (the virus), instead of the standard practices and knowledge of past viral epidemics of the WHO.
- We will not know the implications of these acute changes to infant feeding practices, microbiomes, overall infant morbidity and mortality, maternal health and other unforeseen changes for a long time.

## Introduction

In the challenging circumstances of the COVID-19 pandemic, health workers have required guidance on the clinical management of new mothers with suspected or confirmed COVID-19 and their infants. A key general strategy for preventing the spread of SARS-CoV-2 virus responsible for COVID-19 is the separation of the infected from those uninfected. However, mothers and their infants are a special case as the risk posed by transmission of SARS-CoV-2 must be weighed against the protection afforded infants by proximity and breastfeeding.^1^ For normal health, growth and development, it is recommended that infants initiate breastfeeding within an hour of birth, exclusively breastfed for the first 6 months of life, and continue breastfeeding, along with provision of adequate complementary foods, until 2 years of age or beyond.^2^ The importance of adherence to these breastfeeding practices is well documented with estimates that nearly 700,000 lives are lost annually because recommendations are not universally followed.^3^

Clinical management in the hours and days after birth impacts breastfeeding. Provision of immediate, uninterrupted skin-to-skin (S2S) contact after birth is effective in facilitating early successful initiation of breastfeeding, avoidance of breastmilk substitutes (BMS) and long-term continued breastfeeding.^4^ Rooming-in and maintaining close proximity between mother and newborn also facilitate breastfeeding success, support maternal caregiving capacity and good maternal and infant health outcomes.^5^ In contrast, separation of the mother–newborn dyad, lack of breastfeeding support and early provision of additional foods or fluids, are detrimental to breastfeeding and therefore maternal and infant health.^5^

The understanding of how SARS-CoV-2 and COVID-19 affect pregnant and recently delivered women and infants has exponentially grown in the past 7 months. The transmission of SARS-Co-V-2 due to vaginal birth appears unlikely.^6–9^ While in utero vertical transmission remains unproven,^7 8 10^ there may be some indication of transplacental passage of the virus.^11^ Currently there is no evidence of viable, infective SARS-Co-V-2 in human milk,^12–14^ while there are increasing reports of SARS-Co-V-2 specific immunoglobulin in infected mother’s milk.^14–16^ Isolating infants from their mothers with COVID-19 and prohibiting breastfeeding has not been associated with less postpartum transmission of the virus than where mothers are permitted to breastfeed while using appropriate infection prevention and control (IPC) measures (masks, handwashing, World Health Organization, pp 42–43).^17 18^ As stated by the WHO, ‘In infants, the risk of COVID-19 infection is low, the infection is typically mild or asymptomatic, and the consequences of not breastfeeding or separation of mother and child can be significant’ (p43).^18^

Much of this research has been published in recent months. However, in early March, low rates of serious illness in infants infected with SARS-CoV-2 was already suggested by the evidence.^19–21^ Furthermore, experience with other respiratory viruses indicated it was unlikely that SARS-CoV-2 would be transmitted via breastmilk (World Health Organization, footnote 3).^22^ This early knowledge allowed WHO to publish detailed recommendations on 13 March 2020 on the care of mothers and infants when maternal COVID-19 disease is suspected or confirmed in the *Clinical Management of Severe Acute Respiratory Infection (SARI) when COVID-19 Disease is Suspected: Interim Guidance*.^23^ WHO recommended mothers with suspected or confirmed COVID-19 be supported to practice S2S contact, early and exclusive breastfeeding and rooming-in with their infants while using IPC measures (handwashing and wearing mask) when proximate to their infant.^23^ They also recommended providing psychological support to mothers and infants if they are separated.^23^ On 28 April 2020, WHO published a ‘Question and Answer’ on breastfeeding targeted at health workers clarifying reasons for these recommendations.^24^ Then on 27 May 2020, they published updated interim clinical guidance on COVID-19 in which the recommendations regarding maternal and newborn care were unchanged.^18^ While WHO guidance is suitable for all countries and contexts, governments, organisations and hospitals may develop their own. The aim of our study was to review international governmental and professional medical association COVID-19 guidance on maternal and newborn care to assess alignment with WHO recommendations and the extent to which policy supported or undermined breastfeeding.

## Method

### Design

A critical integrative literature review of international COVID-19 guidance was undertaken. This design was chosen because it ‘summarizes past empirical or theoretical literature to provide a more comprehensive understanding of a particular phenomenon or healthcare problem’ (p546).^25^ In this case, the problem was a lack of knowledge on the degree to which government and professional medical association COVID-19 guidance for breastfeeding and newborn care aligned with WHO recommendations.

### Sample

Sixty-eight country guidance documents from six continents on pregnancy, intrapartum and postpartum care in the context of COVID-19 were reviewed. One guidance document per country (n=33) was included in the analysis. When more than one document per country was identified, a hierarchy of inclusion was followed with national government guidance prioritised, followed by state/provincial government guidance, then professional medical association guidance. When more than one highest hierarchy guidance document from a country was identified, the one judged by author consensus to have the most relevance to hospital staff was included. Wherever possible, country contacts including *Alive* & *Thrive* country staff, Ministry of Health or health worker contacts were asked to confirm that the guidance was used by hospitals. Documents were current at the date of collection and if newer versions were published later, they were not included in the dataset.

### Data collection

International guidance documents on pregnancy, intrapartum and postpartum care in the context of COVID-19 were collected between 21 March and 30 April 2020. Guidance was primarily located through direct request to individuals known to the authors in government and non-government organisations and provided in response to requests made on social media. Web searches were conducted in Google using the search terms of the name of the country, ‘ministry of health’, ‘COVID-19’, ‘guidelines’ and ‘pregnancy’. It was understood that the nature of COVID-19 as an emergency meant that guidance would be published on governmental or organisational websites or emailed directly to health professionals rather than being published in medical journals, therefore a search of medical databases was not undertaken. As many guidance documents as could be located during the time frame were included in the analysis. Where necessary, translation of guidance was undertaken by *Alive & Thrive* staff or by other individuals working in maternal and infant health known to the authors. Where guidance stated that infants should be fed according to standard guidelines, national infant feeding guidelines were referred to and included in the analysis.

### Data analysis

Each guidance document was initially assessed and coded for alignment with the WHO *Clinical Management of Severe Acute Respiratory Infection (SARI) when COVID-19 Disease is Suspected: Interim Guidance, 13 March 2020* 23 by one of the authors (DVH or KG). Each was then coded again by both JC and KM separately. Any discrepancies were discussed by the group and decided by consensus.

The process of coding guidance involved reading each document to identify where text referred to recommendations on the care of infants whose mothers were suspected or confirmed as having COVID-19. Recommendations were coded regarding: (1) S2S contact; (2) early initiation of breastfeeding (EIBF); (3); rooming-in; (4) direct breastfeeding (BF); (6) provision of expressed breastmilk (EBM); (6) provision of donor human milk (DHM); (7) wet nursing (WN); (8) provision of BMS; (9) psychological support for separated mothers (PS-M); and (10) psychological support for separated infants (PS-I). The practices of S2S contact, EIBF and direct BF were coded as recommended when guidance was unambiguously supportive of the practice. Where S2S contact, EIBF and direct BF were supported only on family request and after a discussion of risk, they were coded as not recommended with notation made on the circumstances under which the practice was supported. Where it was recommended that infants be isolated from their mothers, S2S contact, EIBF and direct BF were assumed impossible and coded as not recommended. For coding rooming-in, the Baby-Friendly Hospital Initiative definition requiring that infants remain proximate to their mothers, sharing a bed, in a side-car attached to her bed, or a crib directly beside her bed was used.^26^ Recommendations allowing for mothers to room share with infants at a distance were coded as not recommending rooming-in with notation on the recommendation for physical distancing. Alternate feeding methods were coded based on whether recommendations for use prioritised breastmilk options.

Recommendations regarding use of EBM were coded as following recommended practice where guidance was unambiguously supportive if mothers were not directly breastfeeding. If use of EBM was conditionally supported, it was coded as not following recommended practice with notation on reasons. Recommendations regarding DHM were coded as following recommended practice where guidance supported use when maternal breastfeeding or EBM were unavailable. Recommendations regarding use of BMS were coded as following recommended practice when they specified that use was supported if maternal EBM was unavailable. Recommendations for psychological support for separated mothers and infants were coded regardless of reason for separation. Where there was no information about whether a practice was recommended or not, it was coded as absent.

Where an internal conflict in recommendations was identified, the recommendation that most differed from WHO recommendations was coded and the conflict noted. Where guidance had different recommendations based on maternal symptoms, the guidance for mothers who had the most severe symptoms but were still physically capable of infant care was coded. Where conflicts between guidance from the same country were identified through the guidance collection process, they were noted. Where the meaning of a recommendation was unclear, either country contacts were consulted to advise how health workers were interpreting it or ancillary documents (eg, guidance for mothers) were consulted. Frequency of recommendations according to World Bank country income classification (low, lower-middle, upper-middle, high income) were considered. Idiosyncratic non-evidence-based recommendations were noted. Reference to guidance documents from other countries within guidance was recorded. Media reports regarding hospital practices were not systematically sought but where they were brought to the attention of the authors, note was made on alignment or divergence from national guidance included in the analysis.

## Results

### Country guidance

Guidance from 23 government agencies and 10 professional medical associations were included in the analysis (table 1) from Asia (13), Oceania (1), North America (4), South America (1), Europe (8) and Africa (6) and published between 8 February and 25 April 2020. There was a large variation between countries on whether practices recommended by WHO were recommended, not recommended/prohibited, or absent (online supplemental table 1). None of the countries or professional organisations whose guidance documents were reviewed recommended all aspects of WHO guidance.

10.1136/bmjnph-2020-000184.supp1 Supplementary data

**Table 1:** Breastfeeding recommendations for mothers with confirmed or suspected COVID-19 (n=33)

Practice	Confirmed COVID-19			Suspected COVID-19		
	Recommended n (%)	Not recommended n (%)	No information n (%)	Recommended n (%)	Not recommended n (%)	No information n (%)
S2S contact	9 (27)	15 (45)	9 (27)	8 (24)	14 (42)	11 (33)
EIBF	7 (21)	13 (39)*	13 (39)	6 (18)	12 (36)*	15 (45)
Direct BF	16 (48)	16 (48)*	1 (3)	14 (42)	15 (45)*	4 (12)

*Includes not recommended, recommended only with family preference and recommended only after mother and infant COVID-19 swab test is negative.

BF, breastfeeding; EIBF, early initiation of breastfeeding; S2S, skin-to-skin.

#### Skin-to-skin contact, early initiation of breastfeeding and direct breastfeeding

For women confirmed or suspected of having COVID-19, almost twice as many recommended against S2S contact as recommended for it (table 1). EIBF was not often recommended for women confirmed or suspected of having COVID-19 (table 1). Additionally, often no information regarding S2S contact and EIBF for women with confirmed or suspected COVID-19 was provided (table 2). Direct BF was more commonly recommended than either of these practices for women with both confirmed and suspected COVID-19 (table 1).

**Table 2:** Recommendations on maternal–infant proximity for infants of mothers with confirmed or suspected COVID-19 (n=33)

Maternal–infant proximity	Confirmed n (%)	Suspected n (%)
Rooming-in*	12 (36)	11 (33)
Rooming-in only on family request/preference	3 (9)	3 (9)
Room sharing supported, infant >2 m distant^†^	6 (18)	4 (12)
Rooming-in allowed on negative swab COVID-19 test for mother and infant^‡^	–	6 (18)
Infant and mother isolated from one another	9 (27)	3 (9)
No information provided	3 (9)	6 (18)

*Unrestricted.

†Includes where room sharing is supported with 2 m distance or with 2 m distance and family request.

‡Includes where rooming in is allowed on negative swab test; on negative swab test with family preference; or on negative swab test with 2 m distance and family request.

#### Maternal proximity

Guidance recommended different degrees of maternal–infant proximity for women with confirmed or suspected COVID-19, ranging from rooming-in, to rooming-in or room sharing if the family requests (with risks discussed), room sharing with the infant kept 2 m distance from the mother, to complete isolation of infant and mother (table 2). Guidance on maternal proximity was absent in some documents (table 2). In four documents (Ireland,^27^ Japan,^28^ Mexico^29^ and Spain^30^) placing infants room sharing with their mother in an isolette/incubator was recommended.

#### Alternate feeding methods

In most guidance, when mothers and infants are separated or direct BF is not recommended because of maternal COVID-19 status, provision of EBM to infants was recommended (table 3). A small number of guidance recommended against feeding EBM from mothers with confirmed COVID-19 (table 3). The alternate feeding of DHM and use of BMS were commonly absent from guidance documents. DHM was rarely recommended and none of the countries that recommended against EBM feeding when mothers had COVID-19, recommended DHM. The feeding of BMS was identified as an option for the infants of women with confirmed or suspected COVID-19 when maternal milk was unavailable in less than a quarter of guidance documents (table 3). Only the Canadian guidance^31^ addressed WN and recommended against the practice.

**Table 3:** Recommendations on feeding options for infants of mothers with confirmed and suspected COVID-19 unable to directly breastfeed and recommendations on psychological support for separated mothers and infants (n=33)

Practice	Confirmed COVID-19			Suspected COVID-19		
	Recommended n (%)	Not recommended n (%)	No information n (%)	Recommended n (%)	Not recommended n (%)	No information n (%)
Provision of EBM where infant and mother separated	24 (73)	4 (12)*	5 (15)	23 (70)	3 (9)*	7 (21)
DHM where maternal milk unavailable	4 (12)	0 (0)	29 (88)	3 (9)	0 (0)	30 (91)
WN where maternal milk unavailable	0 (0)	1 (3)	32 (97)	0 (0)	1 (3)	32 (97)
BMS where maternal milk unavailable	8 (24)	0 (0)	25 (76)	7 (21)	0 (0)	26 (79)
PS-M†	6 (18)	0 (0)	27 (82)	–	–	–
PS-I†	2 (6)	0 (0)	31 (94)	–	–	–

*Includes ‘no’ and ‘allowed on condition that the swab COVID-19 test results of mother (and infant) are negative’ and ‘family preference with informed decision making’.

†Includes psychological support whether mothers are suspected or confirmed as having COVID-19.

BMS, breastmilk substitutes; DHM, donor human milk; EBM, expressed breastmilk; PS-I, psychological support for separated infants; PS-M, psychological support for separated mothers; WN, wet nursing.

#### Psychological support

While no guidance recommended against psychological support for separated mother–newborn dyads, few recommended for psychological support for separated mothers and less for separated infants (table 3). No guidance document that recommended isolation of infants from mothers with confirmed or suspected COVID-19 recommended psychological support be provided to either.

#### Recommended practices by infant mortality rate and country economic grouping

Of the 33 countries included in our study, 3 are in the World Bank low-income, 8 in lower-middle income, 7 in upper-middle income and 14 in high-income groupings (table 4). As expected, infant mortality rate (IMR) inversely followed income level with highest IMR associated with the lowest income level and lowest IMR with highest income group (table 4).

**Table 4:** 2019 IMRs and alignment with WHO recommendations by World Bank economic country groupings of per capita income as of 1 July 2020

Low-income economies	* WHO recs met	IMR M (SD) = 40.7 (11.9)	Lower-middle income economies	* WHO recs met	IMR M (SD) = 35.4 (18.5)	Upper-middle-income economies	* WHO recs met	IMR M (SD) = 11.1 (6.6)	High-Income economies	* WHO recs met	Imr M (SD) = 3.4 (0.6)
Burkina Faso	3	54	Bangladesh	7	26	Brazil	2	12	Australia	5	3
Ethiopia	0	37	Côte d'Ivoire	2	59	China	0	7	Canada	8	4
Malawi	6	31	India	1	28	Indonesia	1	20	France	1	4
			Kenya	5	32	Jamaica	2	12	Germany	5	3
			Myanmar	2	36	Malaysia	0	7	Ireland	1	3
			Nepal	5	26	Mexico	3	12	Italy	1	3
			Nigeria	3	74	Thailand	0	8	Japan	1	2
			Philippines	1	22				Norway	6	2
			Vietnam	2	16				Portugal	0	3
									Saudi Arabia	1	6
									Singapore	0	2
									Spain	2	3
									UK	3	4
									USA	1	6

World Bank Country and Lending Groups as of 1 July 2020; low-income economies = US$ 1035 or less; lower-middle income economies = US$ 1036 to US$ 4045; upper-middle-income economies = US$ 4046 to US$ 12 535; high-income economies = US$12 536 or more; https://datahelpdesk.worldbank.org/knowledgebase/articles/906519-world-bank-country-and-lending-groups.

IMR (per 1000 live births) 2019 data, https://data.worldbank.org/indicator/SP.DYN.IMRT.IN.

*WHO recs met = number of WHO recommendations met in country guidance document analysed of the nine possible recommendations: S2S contact; EIBF; rooming-in; direct BF; expressed mother’s own milk; DHM; WN; PS-M; PS-I.

DHM, donor human milk; IMR, infant mortality rate; PS-I, psychological support for separated infants; PS-M, psychological support for separated mothers; S2S, skin-to-skin; WN, wet nursing.

There was significant variability in alignment with WHO recommendations within country income category and neither low country income nor high IMR predicted a high level of alignment with WHO recommendations (table 4). For example, Ethiopia, a low-income country with a high IMR, recommended against or did not mention all nine of the WHO recommendations, while Bangladesh, a lower-middle income country with a high IMR included seven of the nine recommendations. Similarly, Canada, a high-income country with a low IMR had the guidance most aligned with WHO of all the countries we examined, only differing in recommending against WN. Portugal and Singapore, both high-income economies, did not include any WHO recommendations in their guidance documents, and n=6 (43%) of the countries in this category only included one (table 4). The nine specific WHO recommendations by World Bank country economic group are shown in table 5.

**Table 5:** Unconditional support for breastfeeding supportive practices for mothers with confirmed COVID-19 by World Bank country groups as proxies for IMR (n=33)

	Low-income economies ($ 1035 or less) n*=*3	Lower-middle income economies (US$ 1036 to US$ 4045) n*=*9	Upper-middle-income economies (US$ 4046 to US$ 12 535) n*=*7	High-income economies (US$12 536 or more) n*=*14
IMR M (SD)	40.7 (11.9)	35.4 (18.5)	11.1 (6.6)	3.4 (0.6)
Practice	n (%)	n (%)	n (%)	n (%)
S2S contact	1 (33.3)	4 (44.4)	0	4 (28.6)
EIBF	1 (33.3)	3 (33.3)	0	3 (21.4)
Rooming-in	2 (66.7)	4 (44.4)	1 (14.3)	5 (35.7)
Direct BF	2 (66.7)	6 (66.7)	3 (42.9)	6 (42.9)
Use of mother’s expressed milk	2 (66.7)	8 (88.9)	3 (42.9)	11 (78.6)
Use of DHM	0	1 (11.1)	1 (14.3)	2 (14.3)
PS-M	1 (33.3)	2 (22.2)	0	3 (21.4)
PS-I	0	1 (11.1)	0	1 (7.1)

IMR per 1000 live births.

BF, breastfeeding; DHM, donor human milk; EIBF, early initiation of breastfeeding; IMR, infant mortality rate; PS-I, psychological support for separated infants; PS-M, psychological support for separated mothers; S2S, skin-to-skin.

#### Guidance documents referenced

In the country guidance analysed (n=33), the most frequently referenced guidance documents in order of publication date were: (1) the Chinese Expert Consensus on the *Perinatal and Neonatal Management for the Prevention and Control of the 2019 Novel Coronavirus Infection* (China Consensus guidance)^32^ (published 6 February 2020, n=6, 18%); (2) the US Centers for Disease Control *Interim Considerations for Infection Prevention and Control of Coronavirus Disease 2019* (*COVID-19) in Inpatient Obstetric Healthcare Settings* (USCDC guidance)^33^ (published 18 February 2020, n=9, 28% of n=32); (3) the American College of Obstetrics and Gynecology, *Novel Coronavirus 2019* (*COVID-19): Practice Advisory* (ACOG guidance)^34^ (first published 25 February 2020, n=6, 18%); (4) the Royal College of Obstetricians and Gynecologists, Royal College of Midwives, and Royal College of Pediatrics and Child Health (UK), *Coronavirus (COVID-19) Infection in Pregnancy: Information for Healthcare Professionals* (RCOG guidance)^35^ (first published 9 March 2020, n=12, 38% of n=32) and (5) the WHO *Clinical Management of Severe Acute Respiratory Infection (SARI) when COVID-19 Disease is Suspected: Interim Guidance* (WHO guidance)^23^ (published 13 March 2020, n=7, 21%). The ACOG guidance^34^ was an explicit reiteration of the USCDC guidance. Excluding the USA, n*=*13 out of n*=*32 guidance documents cited the USCDC guidance or its reiteration in the form of the ACOG guidance or both.

#### Confusion and conflicts within guidance

Confusion and conflicts within guidance documents were not uncommon. For example, the Nigerian Ministry of Health vacillated between supporting and recommending against maternal proximity stating, ‘*Universal isolation of the infant from either confirmed or suspected infection in the mother is not recommended. However, depending on availability of resources the infant may be separated from the mother until isolation precautions for the mother can be formally discontinued. Based on available evidence, continue with: Skin to skin contact with mother…Breastfeeding is encouraged and supported*’ (pp 6–7).^36^ Some of the confusion could be attributed to a lack of international consensus. For example, the Royal College of Obstetricians and Gynaecologists of Thailand expressed difficulty making a firm recommendation on breastfeeding stating, ‘*Although there is no infection found in breastmilk, there are conflicting reports on whether it should be given. Can children breastfeed? The Royal College of Obstetricians and the World Health Organization take into account the benefits of breastfeeding in the form of bonding. It is recommended that mothers breastfeed if they want … but the Centers of Disease Control recommends that mothers just express their breastmilk…We do not have enough information to support or oppose breastfeeding*’ (p10).^37^ In another example, the Mexican Institute of Perinatology stated that mothers with suspected or confirmed COVID-19 should be isolated from their infants (Section 12), but in the same document, S2S contact and rooming-in are recommended (Section 20).^29^ Idiosyncratic non-evidence-based recommendations included from the Indonesian Society of Obstetrics and Gynecology suggested that infants of mothers with COVID-19 wear face shields during breastfeeding (p3).^38^ In another example, the Burkina Faso Society of Paediatrics and Society of Gynecologists and Obstetricians recommended that mothers with COVID-19 not caress their infants (p17).^39^

Finally, guidance from different organisations within a country were in conflict. The Côte d’Ivoire National Mother and Child Health Programme^40^ recommended against S2S contact and allowed for EIBF only with maternal preference. However, the Côte d’Ivoire National Nutrition Programme recommended immediate S2S contact and EIBF regardless of COVID-19 status.^41^

Instances of hospitals not following national guidance were identified. While the Nepalese Ministry of Health and Population^42^ recommended S2S contact, EIBF, rooming-in and direct BF, a director of a maternity hospital in Kathmandu stated that isolation of infants from mothers with COVID-19 symptoms was hospital policy.^43^ The Madrid newspaper *Elmundo* 44 reported on the birth of twins to a woman with COVID-19 who was supported to have immediate S2S contact, EIBF, and rooming-in with her infants despite the Spanish government recommending against all of these practices.^30^ While the Vietnamese Ministry of Health^45^ recommended against S2S contact, EIBF, and rooming-in, Quanh Ninh Obstetrics and Pediatrics Hospital in Quang Ninh Province implemented a policy that mothers and infants be cared for in line with the WHO guidance (box 1).

Box 1 **Case report of a woman with suspected COVID-19 giving birth to twins in Quang Ninh, Vietnam.**

**Figure 1:**
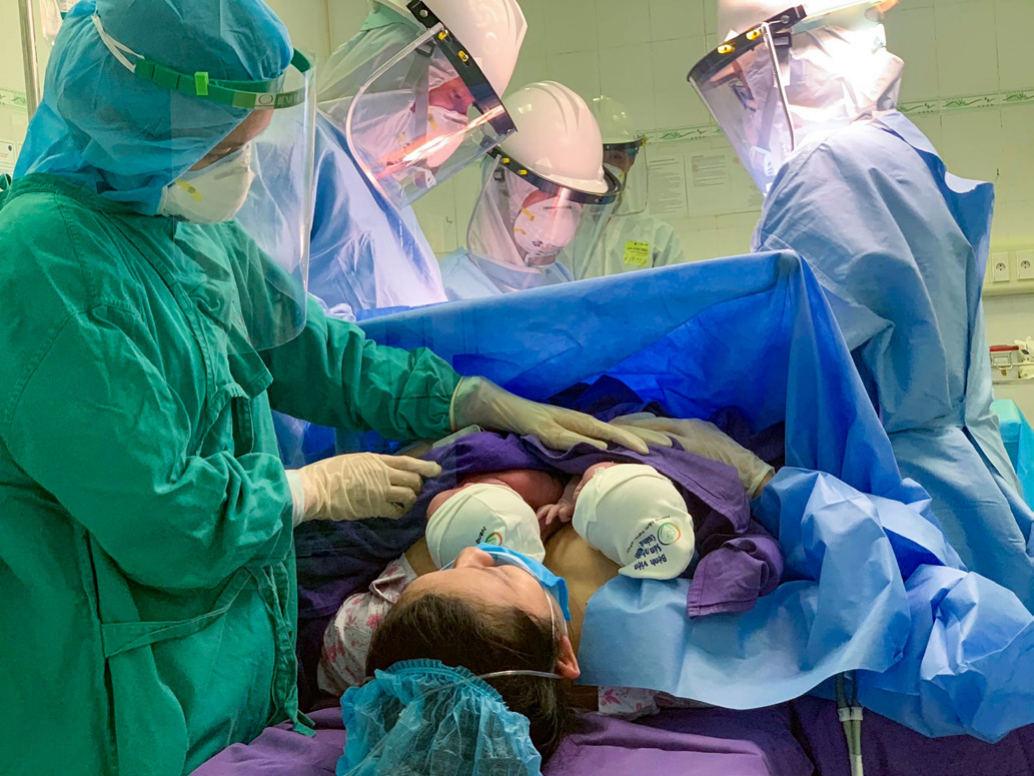
A mother with suspected COVID-19 holding her babies in skin-to-skin contact after caesarean birth at Quang Ninh Obstetrics and Pediatrics Hospital, Vietnam.

On 7 April 2020, a 30-year-old woman arrived in labour at Quang Ninh Obstetrics and Pediatrics Hospital in Northeastern Vietnam. She had been in quarantine since 1 April, after returning from Hanoi, a ‘hotspot’ for COVID-19. Based on her travel, she was deemed a person under investigation for COVID-19 and transferred to the hospital isolation area containing a designated clinic, labour room, operation room, and four postpartum rooms. A pharyngeal sample for Reverse Transcriptase-Polymerase Chain Reaction (RT-PCR) SARS-CoV-2 was sent for testing. A caesarean birth based on multiple pregnancy occurred with all personnel wearing personal protective equipment. The first newborn was dried, followed by delayed cord-clamping, and then placed in skin-to-skin contact with their mother by the midwife. The same early essential newborn care protocol was repeated for the second newborn (figure 1). Remaining in skin-to-skin contact with the mother, the infants completed their first breastfeeds at around 70 and 80 min after birth. Mother and newborns were then transferred to the separate post-operation room and monitored for 6 hours before being moved to a postpartum room. The mother’s first RT-PCR test for SARS-CoV-2 was negative. Mother and newborns roomed-in together, with her husband present as a part of infection control and to assist in caring for the newborns. Counselling on breastfeeding with infection protection and control measures, recognising feeding cues, and the risks of feeding bottles, teats and pacifiers was provided. Both twins were exclusively breastfed while in hospital with the mother wearing a face mask, washing her hands before and after each feed and wiping surfaces. The mother said she felt safe and secure in close contact with her newborns and with the support of her husband. On 13 April 2020, the mother’s second RT-PCR was negative for SARS-CoV-2. The family was discharged the next day, after 14 days of quarantine. The mother was provided with a hotline number for the hospital’s *Little Sun* infant and young child feeding counselling clinic. This case received media attention as this was one of the first women with suspected COVID-19 to give birth in Vietnam.

## Discussion

This analysis of country guidance on breastfeeding and newborn care in the context of COVID-19 revealed considerable variability in recommendations related to S2S contact, EIBF, maternal proximity, direct BF, alternate methods of feeding and psychological support for separated mothers and infants. Irrespective of IMR, guidance recommended against S2S contact, direct BF or supported separation or isolation of infants from their mothers with suspected or confirmed COVID-19. The cost of these recommendations predicts a burden of increased morbidity and mortality due to other infectious causes that will be most evident in low and middle-income countries.^3^ However, even in high-income countries, hospitalisation rates for non-breastfed infants are elevated and policies reducing breastfeeding rates will have an individual and population-based impact on maternal and infant health^46–48^ the effects of which, on top of the effects of this pandemic, we can only postulate at this point. The normal physiology of lactation depends on newborns initiating breastfeeding in the first hour after birth and continuing the frequent neuroendocrine messaging between the infant suckling at the breast and the release of hormones of lactation from the maternal hypothalamic–pituitary axis. Separating the dyad, denying the physiologic stimulation of S2S contact and frequent breastfeeding in this critical period after birth can undermine this process. Their ability to successfully breastfeed may never recover, an obvious harm with long-term effects, manifesting initially by decreased initiation, exclusivity, and duration.^49^

The absence of recommendations for psychological support for separated mothers and infants in the vast majority of guidance is alarming and cannot be justified on the basis of infection control risk.^50^ Physical distance between mothers and infants and lack of breastfeeding undermine maternal caregiving capacity and place infants at increased risk of poor developmental and psychological outcomes, abuse and neglect.^5^ Hynan^51^ highlighted the need for perinatal psychological support in the COVID-19 pandemic, noting the very high rates of post-traumatic stress disorder in parents of infants in neonatal intensive care units where access, S2S contact, and breastfeeding were restricted. Similarly, Morsch and colleagues^50^ described the negative psychological impact of maternal separation on infants and emphasised the importance of emotional care for separated infants in the pandemic. The almost absolute absence of psychological support recommendations for separated mothers and infants suggests a knowledge gap regarding the importance of breastfeeding and maternal proximity for infant well-being that needs to be addressed.^5^ As serious illness due to COVID-19 appears rare in infants,^10^ it may be that hospital practices intended to be protective against COVID-19 present an iatrogenic harm that is of greater risk than infection with SARS-CoV-2.^52^

The full range of alternate feeding options for infants whose mothers were unable to breastfeed them was not recommended in any guidance. Guidance was most clear in support of expressed maternal milk when mothers were unable to directly breastfeed. However, DHM and BMS were less frequently addressed and WN was not recommended at all. The commonality of recommendations for maternal expressed milk where direct breastfeeding was not permitted or possible is encouraging. However, the infrequency with which DHM was addressed, particularly in countries with milk banks, is concerning. Before the pandemic, the Oxford-PATH Human Milk Working Group emphasised the need to establish governance mechanisms and enact legislation for the safe and ethical use of DHM.^53^ During the pandemic, a call to action by the Global Virtual Communication Network of Human Milk Banks and Associations concluded that milk banks are chronically under-resourced and deserve better protection in emergencies.^54^ Unfortunately, implementation guidelines for human milk banks have not been endorsed by the World Health Assembly and few countries have adopted national guidelines and allocated appropriate resources to milk banking. The lack of national and international milk banking guidelines may be partly responsible for BMS superseding DHM in COVID-19 guidance. Exploitation of the pandemic by manufacturers and distributors of BMS, bottles and teats, including promotion to health professionals and the donation of these products to hospitals have been reported.^55 56^ Given the importance of human milk to infant health, the overlooking of DHM in recommendations is unfortunate and together with policies separating mothers and infants may result in increased, unnecessary and harmful use of BMS.

Clinical guidance exists to assist health providers to provide appropriate healthcare. However, non-evidenced-based recommendations, gaps in guidance and conflicts within and between guidance, as identified in this research, present a barrier to provision of appropriate care.^57^ Conflicts between international COVID-19 guidance were confusing to those developing country-specific guidance and in some cases prevented the making of recommendations on breastfeeding,^37^ and resulted in incompatible recommendations within guidance^29^ as we discussed. Idiosyncratic, non-evidence-based recommendations including that infants should breastfeed while wearing a paediatric face shield^38^ or that mothers not caress their infants,^39^ are likely impossible to enforce (and certain to adversely impact breastfeeding and bonding if attempted). Unclear, complex or impractical recommendations do not allow for guidance uptake or consistent provision of care.^57^ Guidance that is not evidence-based risks causing harm. The lack of clear communication, and a poor evidence-base for guidance may be responsible for conflicts between hospital practice and national guidance. In the examples of hospital practice we identified, practice contrary to national guidance worked against maternal and infant well-being in Nepal^42^ but towards better outcomes in Spain^30^ and Vietnam.^45^ In Spain^30^ and Vietnam,^45^ it appeared the hospitals of concern trusted the WHO recommendations over their own government’s guidance. Yeo and colleagues^58^ assessed the rigour of guidance development regarding infants born to mothers with COVID-19 from 17 countries and identified that guidance was developed with limited evidence and was of variable, low methodological rigour. Lack of clarity and conflicts within guidance as well as non-evidence-based recommendations identified by our research may be reflective of this lack of rigour. The evidence-base behind guidance and the guidance itself must be clearly communicated so that clinicians have the confidence to follow it.

The five documents commonly cited by the country guidance included in our study were the China Consensus guidance,^32^ the USCDC guidance,^33^ the ACOG guidance,^34^ the RCOG guidance^35^ and the WHO guidance^23^ published between 6 February and 13 March 2020. These most commonly cited guidance documents can be divided into two groups: guidance that recommends separation of mothers and infants and prohibition of or impediments to breastfeeding (China Consensus,^32^ USCDC,^33^ ACOG^34^) and guidance that recommends mothers and infants be kept together with breastfeeding explicitly supported (WHO^23^ and RCOG^35^). The first two principles of crisis and emergency communications are: be first and be right, as it is recognised that the first source of information often becomes the preferred source and accuracy is necessary to maintain credibility and enable good outcomes.^59^ However, collecting and identifying data to inform recommendations takes time, and there is tension between providing guidance early and providing reliable guidance. Countries with limited resources may rely heavily on recommendations from elsewhere in development of their guidance. However, where sources are unreliable, this can magnify harm. Furthermore, adopting guidance from other countries, or other areas of the world, where the context is different, is risky.sa

The USCDC guidance published on 18 February 2020^33^ was the most influential guidance document in our study, cited by 41% of examined country guidance (inclusive of the ACOG^34^ reiteration and taking into account those that cited both USCDC and ACOG guidance). The USCDC^33^ initially recommended isolation of mothers with COVID-19 from their infants before becoming more supportive of maternal–infant proximity and breastfeeding on 4 April 2020,^60^ then reverting to encouraging maternal–infant separation on 20 May 2020,^61^ and finally changing again to encourage room sharing and breastfeeding on 3 August 2020.^62^ While publishing early may have made the USCDC 18 February 2020 guidance influential, it has not proven reliable. It is unknown whether countries that relied on this USCDC guidance are aware that their recommendations have changed. The USCDC had a similar approach during the H1N1 pandemic where a series of recommendations were made starting with isolation of mothers and infants and avoiding direct breastfeeding and ending with mothers and infants room sharing with direct breastfeeding supported.^63^ The USCDC stated that their initial recommendations regarding maternal and newborn care for the H1N1 and the COVID-19 pandemics were made ‘out of caution’. However, we argue that a cautious approach would value breastfeeding and the development of the maternal–infant relationship and not interrupt either without compelling evidence.^5^

The influence of differential assessment of the value of maternal proximity and breastfeeding in determining recommendations was recognised by WHO, which stated, ‘*WHO’s recommendations on mother/infant contact and breastfeeding are based on a full consideration not only of the risks of infection of the infant with COVID-19, but also the risks of serious morbidity and mortality associated with not breastfeeding or the inappropriate use of infant formula milks as well as the protective effects of skin-to-skin contact and breastfeeding. Recommendations of other organizations may focus only on the prevention of COVID-19 transmission without full consideration of the importance of skin-to-skin contact and breastfeeding*’ (p24).^24^ As was shown in the HIV pandemic, undervaluing the importance of breastfeeding is a mistake that can cost many lives.^1^ In the future, countries should consider organisational reliability and value placed on breastfeeding and the mother–infant relationship in weighing the value of recommendations of others in their guidance development. It is noteworthy that both WHO and RCOG (which is largely in alignment with WHO) were explicit in including an assessment of the importance of breastfeeding and bonding in their guidance development and have not needed to retract recommendations regarding newborn care.

### Study limitations

Guidance from China, Ethiopia, France, Ireland, Italy and the USA were published prior to the release of the WHO guidance and therefore were not able use the WHO recommendations as a guide. We did not assess whether and how country guidance may have changed since collection and did not collect guidance from all countries. Further research to assess whether guidance has been updated based on new evidence and to assess alignment of other country guidance with WHO recommendations is needed.

## Conclusions

Global guidance for breastfeeding and newborn care was poorly aligned with WHO recommendations. The presence of influential guidance conflicting with WHO recommendations and an undervaluing of the importance of maternal proximity and breastfeeding to infant health and maternal caregiving capacity appeared to contribute to this poor alignment. Those developing guidance in the COVID-19 pandemic and other future infectious disease outbreaks need to appropriately consider the importance of S2S contact, EIBF, rooming-in and breastfeeding to maternal and infant physical and psychological health unless there is significant evidence to contraindicate these practices. Additionally, the reliability of expert guidance should be critically appraised based on past performance and priorities. Guidance must be evidence-based, clear and unambiguous. It should also value the importance of DHM where maternal milk is unavailable and psychological support for situations where mothers and infants are unavoidably separated from one another. Recommendations against maternal proximity and breastfeeding should not be made without compelling evidence that it is necessary, and less harmful than maintaining dyad integrity for direct breastfeeding and issues of infant health and maternal caregiving capacity. While the guidance included in this research was published early in the pandemic and may have been revised since to be better aligned with WHO recommendations, many infants and their mothers will have been harmed by policies put in place then, and potentially remaining in place now.

10.1136/bmjnph-2020-000184.supp2 Supplementary data
