# Supplementary material for: Misalignment of global COVID-19 breastfeeding and newborn care guidelines with World Health Organization recommendations
Source: BMJ Nutr Prev Health. 2020 Dec 22;3(2):339–50. doi: 10.1136/bmjnph-2020-000184 (PMC7759756; doi:10.1136/bmjnph-2020-000184)
Supplement: Supplementary data [file bmjnph-2020-000184supp001.pdf]

Supplementary Table 1. Guidance documents included in the review (*N*=33)

Country of origin	Title of document	Type [a]	Date released (2020)	Source
Australia (Victoria)	Coronavirus disease (COVID-19): Maternity and neonatal care guidance for clinicians [1]	GG	23 April	Department of Health and Human Services
Bangladesh	National Guidelines on Clinical Management of Coronavirus Disease 2019 (version 4.0) [2]	GG	30 March	Ministry of Health and Family Welfare
Brazil	NOTA TÉCNICA No 7/2020-DAPES/SAPS/MS [3]	GG	19 March	Ministerio da Saúde
Burkina Faso	Guide de prise en charge de l'infection a COVID-19 chez l'enfant [4]	PG	27 March	Societe Burkinabe de Pediatrie and Société des Gynécologues et Obstériciens du Burkina
Canada	Clinical Management of Patients with Moderate to Severe COVID-19 - Interim Guidance [5]	GG	2 April	Public Health Agency of Canada
China	Notice on strengthening the treatment and safe delivery of maternal diseases during the prevention and control of new coronavirus pneumonia [6]	GG	8 February	National Health Commission of the People's Republic of China
Côte d'Ivoire	Conduite A Tenir En Gynecologie Obstetrique, Neonatalogie En Periode De Pandemie De L'infection A Coronavirus [7]	GG	30 March	Programme National de Santé de la Mère et de l'Enfant
Ethiopia	Case management protocol for Corona Virus Disease-19 (COVID-19) in Ethiopia [8]	GG	1 March	Ministry of Health and Ethiopian Public Health Institute

France	Propositions De La Societe Francaise De Neonatalogie and de la Societe Francaise de Pediatre Concernant Les Nouveau-Nes Dans Le Contexte D'epidemie a COVID-19 [9]	PG	16 March	La Societe Francaise De Neonatalogie
Germany	Empfohlene Präventionsmaßnahmen für die geburtshilfliche Versorgung in deutschen Krankenhäusern und Kliniken im Zusammenhang mit dem Coronavirus [10]	PG	19 March	Deutsche Gesellschaft Fur Gynakologie und Geburtshilfe e.V.
India	Guidance for Management of Pregnant Women in COVID-19 Pandemic [11]	GG	12 April	Indian Council for Medical Research and National Institute for Research in Reproductive Health
Indonesia	Rekomendasi Penanganan Infeksi Virus Corona (COVID-19) Pada Maternal (Hamil, Bersalin Dan Nifas) [12]	PG	18 April	Pokja Infeksi Saluran Reproduksi Perkumpulan Obstetri Dan Ginekologi Indonesia
Ireland	Interim Guidelines on the Management of Suspected COVID-19/SARS-CoV-2 in the pregnant and post partum period [13]	GG	27 February	Health Service Executive Health Protection Surveillance Centre
Italy	Breastfeeding and SARS-CoV-2 Infection (Coronavirus Disease 2019 - COVID-19) Ad interim directions of the Italian Society of Neonatology (SIN) [14]	PG	28 February	Italian Society of Neonatology
Jamaica	Guideline for the management of Pregnancy during the COVID-19 Pandemic [15]	GG	25 March	Ministry of Health and Wellness
Japan	Recommended measures to a new type of coronavirus infection in newborn infants (3rd edition) [16]	PG	23 March	Japanese Society for Neonatal Health
Kenya	Guidelines on the Management of Paediatric Patients during Covid-19 Pandemic [17]	GG	25 March	Ministry of Health

Malaysia	Guidelines on Management of Coronavirus Disease 2019 (Covid 19) in Neonates [18]	GG	24 March	Ministry of Health
Malawi	Infant & Young Child Feeding In The Context Of Covid-19 [19]	GG	N.D.	Ministry of Health
Mexico	Lineamiento técnico: Abordaje de infección por COVID-19 en el periodo perinatal en contingencia [20]	GG	13 April	Instituto Nacional de Perinatología
Myanmar	Clinical management guideline For COVID – 19 infection in pregnancy (version 2/2020) [21]	GG	9 April	Ministry of Health and Sports, Department of Medical Services
Nepal	COVID-19: Guidance for Pregnant Women and Lactating Mothers [22]	GG	N.D.	Ministry of Health and Population
Nigeria	Guidelines for the Management of Pregnant Women and Nursing Mothers [23]	GG	25 April	Federal Ministry of Health and Nigeria Center for Disease Control
Norway	Mødre med symptomer på luftveisinfeksjon og/eller påvist COVID-19-infeksjon: Amming og smitteforebyggende tiltak [24]	GG	20 March	Norwegian Directorate of Health, Norwegian Institute of Public Health
Philippines	Clinical Approach to the Management of COVID-19 in Pregnancy and the Newborn [25]	PG	25 March	Philippine Obstetrical and Gynecological Society
Portugal	COVID-19: Fase de mitigação - Gravidez e Parto [26]	GG	30 March	Divisão de Saúde Sexual Reprodutiva Infantil e Juvenil
Saudi Arabia	COVID-19 in Pregnancy Rapid Response Guidelines Version 1.1 [27]	GG	25 March	Ministry of Health
Singapore	Management of pregnancy and birth in women with coronavirus disease (COVID-19) [28]	PG	20 April	College of Obstetricians and Gynaecologists, Singapore

Spain	Documento técnico: Manejo de la mujer embarazada y el recién nacido con COVID-19 [29]	GG	17 March	Centro de Coordinación de Alertas y Emergencias Sanitarias. Dirección General de Salud Pública, Calidad e Innovación
Thailand	RTCOG Clinical Practice Guideline Management of Covid-19 Infection in Pregnancy [30]	PG	20 March	Royal Thai College of Obstetricians and Gynecologists
United Kingdom	Coronavirus (COVID-19) Infection in Pregnancy Information for healthcare professionals (Version 4.1) [31]	PG	26 March	Royal College of Obstetrics and Gynaecology, Royal College of Midwives, Royal College of Paediatrics and Child Health, Royal College of Anaesthetists, Obstetric Anaesthetists' Association
United States	Interim Considerations for Infection Prevention and Control of Coronavirus Disease 2019 (COVID-19) in Inpatient Obstetric Healthcare Settings [32]	GG	18 February	United States Centers for Disease Control and Prevention
Vietnam	Decision number: 1271 / QD-BYT- Promulgating the guidance on temporary promotion for, and handling of, acute inhabitic infection with SARS-COV-2 (COVID-19) infection in pregnant women and newborn children [33]	GG	21 March	Ministry of Health

Footnote: [a] Type= type of document; GG= government guidance document; PG= professional medical association guidance document; N.D.= no date known

References

1. Department of Health and Human Services Victoria. (2020) *Coronavirus disease (COVID-19) Maternity and neonatal care guidance for clinicians.* Retrieved April 27, 2020, from https://www.dhhs.vic.gov.au/health-services-and-general-practitioners-coronavirus-disease-covid-19
2. Disease Control Division, Directorate General of Health Services, Ministry of Health & Family Welfare, Government of the People's Republic of Bangladesh. (2020). *National Guidelines on Clinical Management of Coronavirus Disease 2019 (COVID-19).* Retrieved April 5, 2020 from https://app.adpc.net/sites/default/files/public/publications/attachments/COVID_Guideline_V4.30.3.2020.pdf
3. Ministério da Saúde do Brasil, Secretaria de Atenção Primária à Saúde Departamento de Ações Programácas Estratégicas. (2020). Nota Técnica No 7/2020-DAPES/SAPS/MS. Retrieved 30 March from http://189.28.128.100/dab/docs/portaldab/documentos/SEIMS_0014033399_Nota_Tecnica.pdf
4. Société Burkinabe de Pediatrie (SOBUPED) & Société des Gynécologues et Obstériciens du Burkina (SOGOB). (2020). *Guide de prise en charge de l'infection a COVID-19 chez l'enfant.*
5. Public Health Agency of Canada. *Clinical management of patients with moderate to severe COVID-19: Interim Guidance April 2, 2020*. Retrieved 15 April 2020 from https://www.ammi.ca/Content/Clinical%20Care%20COVID-19%20Guidance%20FINAL%20April2%20ENGLISH%281%29.pdf

6. National Health Commission of the People's Republic of China. (2020). *Notice on strengthening the treatment and safe delivery of maternal diseases during the prevention and control of new coronavirus pneumonia*. Retrieved 30 March 2020 from http://www.nhc.gov.cn/xcs/zhengcwj/202002/4f80657b346e4d6ba76e2cfc3888c630.shtml
7. Programme National de Santé de la Mère et de l'Enfant de Côte d'Ivoire. (2020). *Conduite a tenir en gynecologie obstetrique, neonatalogie en periode de pandemie de l'infection a coronavirus.* Retrieved 10 April 2020 from http://pnsme.ci/admin/docs/catdvtcascovid19mereenfantadaptdocumentfinal.pdf
8. Ministry of Health-Ethiopia & Ethiopian Public Health Institute. (2020). *Case management protocol for Corona Virus Disease-19 (COVID-19) in Ethiopia - March 2020.* Retrieved 2 April 2020 from https://www.ephi.gov.et/images/novel_coronavirus/PHEOC-_Case-management_COVID-19-_March-2020.pdf
9. Société Française de Néonatalogie. (2020). *Recomandations de la Société Française de Néonatalogie concernant les nouveau- nes dans le contexte d'epidemie à COVID-19.* Retrieved 29 March 2020 from https://www.gynecologie-pratique.com/sites/www.gynecologie-pratique.com/files/recommandations/recommandations-sfn-covid-19.pdf
10. Deutsche Gesellschaft für Gynäkologie und Geburtshilfe e.V. (19 March 2020). *Empfohlene Präventionsmaßnahmen für die geburtshilfliche Versorgung in deutschen Krankenhäusern und Kliniken im Zusammenhang mit dem Coronavirus*. Retrieved 25 March 2020 from https://www.dggg.de/fileadmin/documents/Weitere_Nachrichten/2020/COVID-19_DGGG-Empfehlungen_fuer_Kreissaele_20200319_f.pdf

11. Indian Council of Medical Research – National Institute for Research in Reproductive Health. (2020). *Guidance for Management of Pregnant Women in COVID-19 Pandemic.* Retrieved 22 April 2020 from https://www.icmr.gov.in/pdf/covid/techdoc/Guidance_for_Management_of_Pregnant_Women_in_COVID19_Pandemic_12042020.pdf
12. Perkumpulan Obstetri dan Ginekologi Indonesia. *Rekomendasi Penanganan Infeksi Virus Corona (COVID-19) pada Maternal (Hamil, bersalin dan nifas)*. (2020). Retrieved 22 April 2020 from https://pogi.or.id/publish/wp-content/uploads/2020/03/Rekomendasi-Penanganan-Infeksi-COVID-19-pada-maternal.pdf
13. Health Service Executive Ireland. (2020). *Interim Guidelines on the management of suspected COVID-19/SARS-CoV-2 in the pregnant and post-partum period* V1.0 27.02.2020.
14. Societa Italana di Neonatologia. (2020). *Breastfeeding and SARS-CoV-2 Infection (Coronavirus Disease 2019 - COVID-19) Ad interim directions of the Italian Society of Neonatology (SIN)* Retrieved 10 April 2020 from: https://www.uenps.eu/wp-content/uploads/2020/03/14marzo.SIN_UENPS0.pdf
15. Ministry of Health & Wellness Jamaica. (25 March 2020). *Guideline for the management of pregnancy during the COVID-19 pandemic*. Retrieved 5 April 2020 from https://www.moh.gov.jm/wp-content/uploads/2020/04/Guideline-for-the-Management-of-Pregnancy-During-the-COVID-19-Pandemic.pdf
16. Japanese Society for Neonatal Health and Development. (2020). *Recommended measures to a new type of coronavirus infection in newborn infants.* Retrieved 28 March 2020 from http://jsnhd.or.jp/pdf/202000326COVID-19.pdf

17. Ministry of Health Kenya. (2020). *Guidelines on the Management of Paediatric Patients During COVID-19 Pandemic*. Retrieved 28 March 2020 from https://www.health.go.ke/wp-content/uploads/2020/04/PAEDIATRIC-Covid-Guidelines-Final.pdf

18. Ministry of Health Malaysia. (2020). Guideline on Management of Coronavirus Disease 2019 (COVID-19) In Pregnancy. Retrieved 1 April 2020 from https://www.moh.gov.my/moh/resources/Penerbitan/Garis%20Panduan/Pengurusan%20KEsihatan%20&%20kawalan%20pykit/2019-nCOV/Bil%204%20%202020/Annex%2023%20%20Guidelines%20COVID%20Pregnancy_26022020.pdf

19. Ministry of Health Malawi. (2020). *Infant & young child feeding in the context of COVID-19.*

20. Instituto Nacional de Perinatología. (2020). Lineamiento Tecnico: Abordaje De Infección Por Covid-19 En El Periodo Perinatal En Contingencia. Retrieved 29 April 2020 from http://www.eneo.unam.mx/novedades/coms130420/LineamientoINPerCOVID19.pdf

21. Ministry of Health and Sports, Department of Medical Services. Clinical Management Guideline for COVID-19 Infection in Pregnancy (Version 2/2020) Myanmar. Retrieved 15 April 2020 from http://mohs.gov.mm/su/oxpp6002OX

22. Ministry of Health and Population, Government of Nepal. COVID-19: Guidance for Pregnant Women and Lactating Mothers. (N.D.) Retrieved 30 April 2020 from https://nepal.unfpa.org/en/publications

23. Federal Ministry of Health & Nigeria Centre for Disease Control. (2020). Guidelines for the Management of Pregnant Women and Nursing Mothers. Retrieved 30 April 2020 from https://covid19.ncdc.gov.ng/media/files/GuidelinesforMgtofPregnantWomen_t5skUIm.pdf

24. Norwegian Directorate of Health & Norwegian Institute of Public Health. (2020). Guidance for handling pregnant women with proven or possible COVID-19 infection (coronavirus). Retrieved 10 April 2020 from https://www.legeforeningen.no/foreningsledd/fagmed/norsk-gynekologisk-forening/veiledere/svangerskap-covid19/
25. Philippine Obstetrical and Gynecological Society. (2020). Clinical Approach to the Management of COVID-19 in Pregnancy and the Newborn. Retrieved 8 April 2020 from https://www.pogsinc.org/index.php/meetings/aofog-2019/item/566-approach-to-the-management-of-covid-19-in-pregnancy-and-the-newborn
26. Serviço Nacional de Saúde de Portugal. (2020). Guideline no. 018-2020: COVID-19: FASE DE MITIGAÇÃO - Gravidez e Parto. Retrieved 8 April 2020 from https://www.sns.gov.pt/noticias/2020/03/26/covid-19-fase-de-mitigacao/
27. Ministry of Health Saudi Arabia. (2020). COVID-19 in Pregnancy: Rapid Response Guidelines Version 1.1. Retrieved 4 April 2020 https://www.moh.gov.sa/Ministry/MediaCenter/Publications/Documents/COVID-19-IN-PREGNANCY-MOH-Protocol.pdf
28. Academy of Medicine Singapore & College of Obstetricians & Gynaecologists, Singapore. (2020). Management of Pregnancy and Birth in Women with Coronavirus Disease (Covid-19). Retrieved 25 April 2020 from https://www.ams.edu.sg/view-pdf.aspx?file=media%5C5443_fi_921.pdf&ofile=(Committee+Opinion)+Management+of+Pregnancy+and+Birth+in+Women+with+Covid-19+April+(20200420).pdf
29. Ministerio de Sanidad, Gobierno de Espana. (2020). Documento tecnico: Manejo de la mujer embarazada y el recién nacido con COVID-19. Retrieved 21 March 2020

https://www.areasaludbadajoz.com/docencia_investigacion/lecturas_recomendadas/Documento_manejo_embarazo_recien_nacido.pdf

30. Royal Thai College of Obstetricians and Gynecologists. (2020). Clinical Practice Guideline Management of Covid-19 Infection in Pregnancy. Retrieved April 5, 2020 from http://www.rtcog.or.th/home/cpg-management-of-covid-19-infection-in-pregnancy/3572/
31. Royal College of Obstetrics and Gynaecology, Royal College of Midwives, Royal College of Paediatrics and Child Health, Royal College of Anaesthetists, Obstetric Anaesthetists' Association. (2020). Coronavirus (COVID-19) Infection in Pregnancy: Information for Healthcare Professionals Version 4.1. Retrieved 26 March 2020 from: https://www.righttocare.org/wp-content/uploads/2020/rtc/COVID%2019%20Resources/Training%20for%20Healthcare%20Workers%20PAGE/Guidelines/Clinical%20Guidelines/For%20guidance%20on%20COVID-19%20in%20pregnancy.pdf
32. Centers for Disease Control and Prevention. (2020). Interim Considerations for Infection Prevention and Control of Coronavirus Disease 2019 (COVID-19) in Inpatient Obstetric Healthcare Settings, February 18. Retrieved 21 March 2020 from https://stacks.cdc.gov/view/cdc/85196
33. Ministry of Health Viet Nam. (2020). Decision 1271/QD-BYT dated March 21, 2020 by Ministry of Health on Interim guidance for prevention and management of SARS-CoV-2 (COVID-19) among pregnant women and newborns. Retrieved 23 March 2020 from https://ncov.moh.gov.vn/-/bo-y-te-huong-dan-du-phong-va-xu-tri-covid-19-o-phu-nu-mang-thai-tre-so-sinh
