# Supplementary material for: Misalignment of global COVID-19 breastfeeding and newborn care guidelines with World Health Organization recommendations
Source: BMJ Nutr Prev Health. 2020 Dec 22;3(2):339–50. doi: 10.1136/bmjnph-2020-000184 (PMC7759756; doi:10.1136/bmjnph-2020-000184)
Supplement: Supplementary data [file bmjnph-2020-000184supp002.pdf]

Supplementary Table 2. Alignment of national COVID-19 guidance on breastfeeding and newborn care with WHO recommendations: our data

Countries	COVID-19	S2S	EIBF	RI	BF	EBM	DHM	WN	BMS	PS-M	PS-I
Australia[1]	Suspected	v	0	v	v	v	0	0	0	-	-
	Confirmed	v	0	v	v	v	0	0	0	v	0
Bangladesh[2]	Suspected	v	v	v	v	v	0	0	0	-	-
	Confirmed	v	v	v	v	v	0	0	0	v	v
Brazil[3]	Suspected	0	0	0	v	v	0	0	0	-	-
	Confirmed	0	0	0	v	v	0	0	0	0	0
Burkina Faso[4]	Suspected	x	0	v	v	v	0	0	v	-	-
	Confirmed	x	0	v	v	v	0	0	v	0	0
Canada[5]	Suspected	v	v	v	v	v	v	x	v	-	-
	Confirmed	v	v	v	v	v	v	x	v	v	v
China[6]	Suspected	x	x	x, t	x	x	0	0	0	-	-
	Confirmed	x	x	x	x	x	0	0	0	0	0
Côte d'Ivoire[7]	Suspected	x	x, f	0	v	v	0	0	v	-	-
	Confirmed	x	x, f	0	v	v	0	0	v	0	0

Ethiopia[8]	Suspected	x	x	x	x	0	0	0	0	-	-
	Confirmed	x	x	x	x	0	0	0	0	0	0
France[9]	Suspected	0	0	0	0	0	0	0	0	-	-
	Confirmed	0	0	x, 2	v	0	0	0	0	0	0
Germany[10]	Suspected	v	v	v	v	v	0	0	0	-	-
	Confirmed	v	v	v	v	v	0	0	0	0	0
India[11]	Suspected	0	0	x, 2, f	x, f	v	0	0	0	-	-
	Confirmed	0	0	x, 2, f	x, f	v	0	0	0	0	0
Indonesia[12]	Suspected	x	x	x	v	0	0	0	0	-	-
	Confirmed	x	x	x	v	0	0	0	0	0	0
Ireland[13]	Suspected	x	x	x, f	x, f	v	0	0	0	-	-
	Confirmed	x	x	x, f	x, f	v	0	0	0	0	0
Italy[14]	Suspected	0	0	x, 2, f, t	x, t	v	0	0	v	-	-
	Confirmed	0	0	x, 2, f	x	v	0	0	v	0	0
Jamaica[15]	Suspected	0	0	v	x, f	v	0	0	v	-	-
	Confirmed	0	0	v	x, f	v	0	0	v	0	0
Japan[16]	Suspected	x	x	x, f	x	v	0	0	0	-	-
	Confirmed	x	x	x, f	x	v	0	0	0	0	0

Kenya[17]	Suspected	v	0	v	v	v	0	0	v	-	-
	Confirmed	v	0	v	v	v	0	0	v	v	0
Malaysia[18]	Suspected	x	x	x, t	x, t	x, t	0	0	0	-	-
	Confirmed	x	x	x	x	x	0	0	0	0	0
Malawi[19]	Suspected	v	v	v	v	v	0	0	v	-	-
	Confirmed	v	v	v	v	v	0	0	v	v	0
Mexico[20]	Suspected	x	x	x, 2	v	v	v	0	0	-	-
	Confirmed	x	x	x, 2	v	v	v	0	0	0	0
Myanmar[21]	Suspected	0	0	v	x, f	v	0	0	0	-	-
	Confirmed	0	0	v	x, f	v	0	0	0	0	0
Nepal[22]	Suspected	0	0	0	0	0	0	0	0	-	-
	Confirmed	v	v	v	v	v	0	0	0	0	0
Nigeria[23]	Suspected	v	v	0	v	0	0	0	0	-	-
	Confirmed	v	v	0	v	0	0	0	0	0	0
Norway[24]	Suspected	v	v	v	v	v	0	0	0	-	-
	Confirmed	v	v	v	v	v	0	0	0	v	0
Philippines[25]	Suspected	x	x	x, t	x, f	v	0	0	0	-	-
	Confirmed	x	x	x	x, f	v	0	0	0	0	0

Portugal[26]	Suspected	x	0	x, f	0	x, t	0	0	0	-	-
	Confirmed	x	0	x, f	0	x	0	0	0	0	0
Saudi Arabia[27]	Suspected	x	x	x	x	v	0	0	0	-	-
	Confirmed	x	x	x	x	v	0	0	0	0	0
Singapore[28]	Suspected	0	0	0	0	0	0	0	0	-	-
	Confirmed	x	x	x	x	x	0	0	0	0	0
Spain[29]	Suspected	x	x	x, t	x, f, t	v	v	0	0	-	-
	Confirmed	x	x	x	x, f	v	v	0	0	0	0
Thailand[30]	Suspected	x	x	x, 2, f, t	x	0	0	0	0	-	-
	Confirmed	x	x	x, 2, f	x	0	0	0	0	0	0
United Kingdom[31]	Suspected	0	0	v	v	v	0	0	0	-	-
	Confirmed	0	0	v	v	v	0	0	0	0	0
United States[32]	Suspected	0	0	x, 2, f	x, f	v	0	0	0	-	-
	Confirmed	0	0	x, 2, f	x, f	v	0	0	0	0	0
Vietnam[33]	Suspected	0	0	x, 2	x, f	v	0	0	0	-	-
	Confirmed	0	0	x	x	v	v	0	v	0	0

Note. Variables: S2S=Skin-to-skin contact; EIBF=Early Initiation of Breastfeeding; RI=Rooming-in; BF=Direct breastfeeding with infection protection and control measures; EBM=Expressed milk recommended if direct breastfeeding not available; DHM=Donor Human Milk recommended if maternal milk not available; WN=Wet nursing

recommended if maternal milk not available; BMS=Breastmilk substitutes recommended if breastmilk not available; PS-M= psychological support for mothers where separated ; PS-I= psychological support for infants where separated. Coding: v=Recommended; x= Not Recommended; x, t= Allowed if swab test results of mother are negative; x, f= Allowed based on family preference; x, f, t= Allowed if swab test results of mother are negative and based on family preference; x, 2=Room sharing allowed with two meters distance; x, 2, f=Room sharing allowed with two meters distance upon family request/preference; x, 2, f, t= Room sharing allowed with two meters distance, family preference, and negative swab test.

References

1. Department of Health and Human Services Victoria. (2020) *Coronavirus disease (COVID-19) Maternity and neonatal care guidance for clinicians.* Retrieved April 27, 2020, from https://www.dhhs.vic.gov.au/health-services-and-general-practitioners-coronavirus-disease-covid-19
2. Disease Control Division, Directorate General of Health Services, Ministry of Health & Family Welfare, Government of the People's Republic of Bangladesh. (2020). *National Guidelines on Clinical Management of Coronavirus Disease 2019 (COVID-19).* Retrieved April 5, 2020 from https://app.adpc.net/sites/default/files/public/publications/attachments/COVID_Guideline_V4.30.3.2020.pdf

3. Ministério da Saúde do Brasil, Secretaria de Atenção Primária à Saúde Departamento de Ações Programácas Estratégicas. (2020). Nota Técnica No 7/2020-DAPES/SAPS/MS. Retrieved 30 March from http://189.28.128.100/dab/docs/portaldab/documentos/SEIMS_0014033399_Nota_Tecnica.pdf
4. Société Burkinabe de Pediatrie (SOBUPED) & Société des Gynécologues et Obstériciens du Burkina (SOGOB). (2020). *Guide de prise en charge de l'infection a COVID-19 chez l'enfant.*
5. Public Health Agency of Canada. *Clinical management of patients with moderate to severe COVID-19: Interim Guidance April 2, 2020*. Retrieved 15 April 2020 from https://www.ammi.ca/Content/Clinical%20Care%20COVID-19%20Guidance%20FINAL%20April2%20ENGLISH%281%29.pdf
6. National Health Commission of the People's Republic of China. (2020). *Notice on strengthening the treatment and safe delivery of maternal diseases during the prevention and control of new coronavirus pneumonia*. Retrieved 30 March 2020 from http://www.nhc.gov.cn/xcs/zhengcwj/202002/4f80657b346e4d6ba76e2cfc3888c630.shtml
7. Programme National de Santé de la Mère et de l'Enfant de Côte d'Ivoire. (2020). *Conduite a tenir en gynecologie obstetrique, neonatalogie en periode de pandemie de l'infection a coronavirus.* Retrieved 10 April 2020 from http://pnsme.ci/admin/docs/catdvtcascovid19mereenfantadaptdocumentfinal.pdf
8. Ministry of Health-Ethiopia & Ethiopian Public Health Institute. (2020). *Case management protocol for Corona Virus Disease-19 (COVID-19) in Ethiopia - March 2020.* Retrieved 2 April 2020 from https://www.ephi.gov.et/images/novel_coronavirus/PHEOC-_Case-management_COVID-19-_March-2020.pdf

9. Société Française de Néonatalogie. (2020). *Recomandations de la Société Française de Néonatalogie concernant les nouveau- nes dans le contexte d'epidemie à COVID-19.* Retrieved 29 March 2020 from https://www.gynecologie-pratique.com/sites/www.gynecologie-pratique.com/files/recommandations/recommandations-sfn-covid-19.pdf

10. Deutsche Gesellschaft für Gynäkologie und Geburtshilfe e.V. (19 March 2020). *Empfohlene Präventionsmaßnahmen für die geburtshilfliche Versorgung in deutschen Krankenhäusern und Kliniken im Zusammenhang mit dem Coronavirus*. Retrieved 25 March 2020 from https://www.dggg.de/fileadmin/documents/Weitere_Nachrichten/2020/COVID-19_DGGG-Empfehlungen_fuer_Kreissaele_20200319_f.pdf

11. Indian Council of Medical Research – National Institute for Research in Reproductive Health. (2020). *Guidance for Management of Pregnant Women in COVID-19 Pandemic.* Retrieved 22 April 2020 from https://www.icmr.gov.in/pdf/covid/techdoc/Guidance_for_Management_of_Pregnant_Women_in_COVID19_Pandemic_12042020.pdf

12. Perkumpulan Obstetri dan Ginekologi Indonesia. *Rekomendasi Penanganan Infeksi Virus Corona (COVID-19) pada Maternal (Hamil, bersalin dan nifas)*. (2020). Retrieved 22 April 2020 from https://pogi.or.id/publish/wp-content/uploads/2020/03/Rekomendasi-Penanganan-Infeksi-COVID-19-pada-maternal.pdf

13. Health Service Executive Ireland. (2020). *Interim Guidelines on the management of suspected COVID-19/SARS-CoV-2 in the pregnant and post-partum period* V1.0 27.02.2020.

14. Societa Italana di Neonatologia. (2020). *Breastfeeding and SARS-CoV-2 Infection (Coronavirus Disease 2019 - COVID-19) Ad interim directions of the Italian Society of Neonatology (SIN)* Retrieved 10 April 2020 from: https://www.uenps.eu/wp-content/uploads/2020/03/14marzo.SIN_UENPS0.pdf
15. Ministry of Health & Wellness Jamaica. (25 March 2020). *Guideline for the management of pregnancy during the COVID-19 pandemic*. Retrieved 5 April 2020 from https://www.moh.gov.jm/wp-content/uploads/2020/04/Guideline-for-the-Management-of-Pregnancy-During-the-COVID-19-Pandemic.pdf
16. Japanese Society for Neonatal Health and Development. (2020). *Recommended measures to a new type of coronavirus infection in newborn infants.* Retrieved 28 March 2020 from http://jsnhd.or.jp/pdf/202000326COVID-19.pdf
17. Ministry of Health Kenya. (2020). *Guidelines on the Management of Paediatric Patients During COVID-19 Pandemic*. Retrieved 28 March 2020 from https://www.health.go.ke/wp-content/uploads/2020/04/PAEDIATRIC-Covid-Guidelines-Final.pdf
18. Ministry of Health Malaysia. (2020). Guideline on Management of Coronavirus Disease 2019 (COVID-19) In Pregnancy. Retrieved 1 April 2020 from https://www.moh.gov.my/moh/resources/Penerbitan/Garis%20Panduan/Pengurusan%20KEsihatan%20&%20kawalan%20pykit/2019-nCOV/Bil%204%20%202020/Annex%2023%20%20Guidelines%20COVID%20Pregnancy_26022020.pdf

19. Ministry of Health Malawi. (2020). *Infant & young child feeding in the context of COVID-19.*

20. Instituto Nacional de Perinatología. (2020). Lineamiento Tecnico: Abordaje De Infección Por Covid-19 En El Periodo Perinatal En Contingencia. Retrieved 29 April 2020 from http://www.eneo.unam.mx/novedades/coms130420/LineamientoINPerCOVID19.pdf

21. Ministry of Health and Sports, Department of Medical Services. Clinical Management Guideline for COVID-19 Infection in Pregnancy (Version 2/2020) Myanmar. Retrieved 15 April 2020 from http://mohs.gov.mm/su/oxpp6002OX

22. Ministry of Health and Population, Government of Nepal. COVID-19: Guidance for Pregnant Women and Lactating Mothers. (N.D.) Retrieved 30 April 2020 from https://nepal.unfpa.org/en/publications

23. Federal Ministry of Health & Nigeria Centre for Disease Control. (2020). Guidelines for the Management of Pregnant Women and Nursing Mothers. Retrieved 30 April 2020 from https://covid19.ncdc.gov.ng/media/files/GuidelinesforMgtofPregnantWomen_t5skUIm.pdf

24. Norwegian Directorate of Health & Norwegian Institute of Public Health. (2020). Guidance for handling pregnant women with proven or possible COVID-19 infection (coronavirus). Retrieved 10 April 2020 from https://www.legeforeningen.no/foreningsledd/fagmed/norsk-gynekologisk-forening/veiledere/svangerskap-covid19/

25. Philippine Obstetrical and Gynecological Society. (2020). Clinical Approach to the Management of COVID-19 in Pregnancy and the Newborn. Retrieved 8 April 2020 from https://www.pogsinc.org/index.php/meetings/aofog-2019/item/566-approach-to-the-management-of-covid-19-in-pregnancy-and-the-newborn

26. Serviço Nacional de Saúde de Portugal. (2020). Guideline no. 018-2020: COVID-19: FASE DE MITIGAÇÃO - Gravidez e Parto. Retrieved 8 April 2020 from https://www.sns.gov.pt/noticias/2020/03/26/covid-19-fase-de-mitigacao/

27. Ministry of Health Saudi Arabia. (2020). COVID-19 in Pregnancy: Rapid Response Guidelines Version 1.1. Retrieved 4 April 2020 https://www.moh.gov.sa/Ministry/MediaCenter/Publications/Documents/COVID-19-IN-PREGNANCY-MOH-Protocol.pdf

28. Academy of Medicine Singapore & College of Obstetricians & Gynaecologists, Singapore. (2020). Management of Pregnancy and Birth in Women with Coronavirus Disease (Covid-19). Retrieved 25 April 2020 from https://www.ams.edu.sg/view-pdf.aspx?file=media%5C5443_fi_921.pdf&ofile=(Committee+Opinion)+Management+of+Pregnancy+and+Birth+in+Women+with+Covid-19+April+(20200420).pdf

29. Ministerio de Sanidad, Gobierno de Espana. (2020). Documento tecnico: Manejo de la mujer embarazada y el recién nacido con COVID-19. Retrieved 21 March 2020 https://www.areasaludbadajoz.com/docencia_investigacion/lecturas_recomendadas/Documento_manejo_embarazo_recien_nacido.pdf

30. Royal Thai College of Obstetricians and Gynecologists. (2020). Clinical Practice Guideline Management of Covid-19 Infection in Pregnancy. Retrieved April 5, 2020 from http://www.rtcog.or.th/home/cpg-management-of-covid-19-infection-in-pregnancy/3572/

31. Royal College of Obstetrics and Gynaecology, Royal College of Midwives, Royal College of Paediatrics and Child Health, Royal College of Anaesthetists, Obstetric Anaesthetists' Association. (2020). Coronavirus (COVID-19) Infection in Pregnancy: Information for Healthcare Professionals Version 4.1. Retrieved 26 March 2020 from: https://www.righttocare.org/wp-content/uploads/2020/rtc/COVID%2019%20Resources/Training%20for%20Healthcare%20Workers%20PAGE/Guidelines/Clinical%20Guidelines/For%20guidance%20on%20COVID-19%20in%20pregnancy.pdf

32. Centers for Disease Control and Prevention. (2020). Interim Considerations for Infection Prevention and Control of Coronavirus Disease 2019 (COVID-19) in Inpatient Obstetric Healthcare Settings, February 18. Retrieved 21 March 2020 from https://stacks.cdc.gov/view/cdc/85196

33. Ministry of Health Viet Nam. (2020). Decision 1271/QD-BYT dated March 21, 2020 by Ministry of Health on Interim guidance for prevention and management of SARS-CoV-2 (COVID-19) among pregnant women and newborns. Retrieved 23 March 2020 from https://ncov.moh.gov.vn/-/bo-y-te-huong-dan-du-phong-va-xu-tri-covid-19-o-phu-nu-mang-thai-tre-so-sinh
